# Implications of Lessons Learned From Tobacco Control for Tanning Bed Reform

**DOI:** 10.5888/pcd10.120186

**Published:** 2013-02-28

**Authors:** Craig Sinclair, Jennifer K. Makin

**Affiliations:** Author Affiliation: Craig Sinclair, Cancer Council Victoria, Carlton, Victoria, Australia.

## Abstract

Tanning beds used according to the manufacturer’s instructions expose the user to health risks, including melanoma and other skin cancers. Applying the MPOWER model (monitor, protect, offer alternatives, warn, enforce, and raise taxes), which has been used in tobacco control, to tanning bed reform could reduce the number of people at risk of diseases associated with tanning bed use. Among the tactics available to government are restricting the use of tanning beds by people under age 18 and those with fair skin, increasing the price of tanning bed services through taxation, licensing tanning bed operators, and banning unsupervised tanning bed operations.

## Introduction

In 2009, the International Agency for Research on Cancer (IARC) elevated use of ultraviolet radiation (UVR)-emitting tanning beds to the highest cancer risk category, labeling them “carcinogenic to humans” (1). This means, in effect, that tanning beds are one of the few industries whose product, if used as the manufacturer intends, puts the user at risk of harm. Another industry in which this is the case is the tobacco industry. In the United States alone, according to tanning bed industry estimates, approximately 18,000 small businesses employing more than 120,000 people serve 30 million customers each year (www.theita.com). Although this is small in comparison to the tobacco industry, many lessons learned in the fight against the tobacco industry could be applied to reducing use of tanning beds.

## Similarities Between Tobacco and Tanning Bed Industries

The tobacco and tanning bed industries share several key similarities. The products of both industries cause negative health outcomes. For both tobacco and tanning beds, using the product according to the manufacturer’s instructions can harm the user. For tobacco, smoking is the cause of more than 10% of premature deaths worldwide (2). Exposure to UVR in a tanning bed increases the risk of melanoma and other skin cancers. A recent systematic review estimated that tanning bed use before age 35 increases the risk of melanoma by 87% and demonstrated a dose–response relationship between tanning bed use and melanoma risk (3). Although there are no estimates of worldwide incidence and mortality, in the United Kingdom alone an estimated 100 deaths per year occur from tanning bed use (4). In Australia, tanning bed use accounts for an estimated 281 new melanomas, 43 melanoma-related deaths, and 2,572 new cases of squamous cell carcinoma per year (5), and causes 1 in 6 melanomas in people aged 18 to 29 (6).

Both the tobacco and tanning bed industries provide a product or service that can be addictive (7,8). That tobacco use is addictive is well-established (7). However, many frequent tanners show signs and symptoms of addiction similar to those used as criteria for substance abuse or dependence, including withdrawal symptoms (8). Tanning may also have mood-enhancing effects arising from either biochemical mechanisms or psychological dependence on exposure to UVR.

The tobacco and tanning bed industries both support third-party advocacy groups and lobbyists to advance their interests. For example, tobacco companies have used litigation, direct lobbying to governments, and the establishment of industry-based advocacy bodies to influence not only policy makers and politicians but also public opinion. Industry tactics include political donations, mobilizing protobacco lobby groups in the community, and financing “independent expert” spokespeople (9). The tanning bed industry has also established or supported advocacy groups, including the Indoor Tanning Association (www.theita.com), Tanning Truth (www.tanningtruth.com), the Ultraviolet Foundation (uvfoundation.org), and the Vitamin D Council (vitamindcouncil.org).

Both industries have employed remarkably similar advertising strategies, designed to counteract health concerns associated with their services and appeal to a sense of social popularity and acceptance (10). Tobacco advertising is now banned in the United States, Australia, and many other countries. Historically, however, both tobacco and tanning bed advertising have focused on mitigating health concerns: for example, they use physicians as spokespeople to emphasize alleged health advantages and appeal to a sense of social popularity including using idealized depictions of users and celebrity endorsements (10). Both industries have targeted young people by appealing to image-based social norms and by cost-reduction promotional strategies that may be particularly appealing to young people (eg, selling individual cigarettes or discount deals for multiple tanning sessions).

Given these similarities and the considerable research that has gone into evaluating tobacco control initiatives, skin cancer prevention advocates could learn many lessons from the coordinated public health interventions that have reduced smoking prevalence in Western societies. To assist in the country-level implementation of effective interventions to reduce the demand for tobacco, the World Health Organization developed the MPOWER model (monitor, protect, offer alternatives, warn, enforce, and raise taxes) (www.who.int/tobacco/mpower/en/). The purpose of this article is to explore the potential for applying some of MPOWER’s principles to reduce tanning bed use.

## Applying the MPOWER Model to Tanning Bed Reform

### Monitor tanning bed use and prevention policies

Managing and evaluating interventions to reduce tanning bed use entails monitoring the size of the industry, the number and type of people who use tanning beds, the frequency of use, and compliance of tanning salons with any existing industry standards or legislation. In jurisdictions where industry standards or legislation restrict minors’ access to tanning beds, compliance monitoring should be sufficiently rigorous to detect infractions. Lessons learned from monitoring youth access to cigarettes can be applied to such compliance monitoring. For example, in a study of 276 youth smokers in California, 71% reported lying about their age to purchase cigarettes (11). Researchers found that sales of cigarettes to minors were higher when minors misreported their ages to shop assistants (12). California has monitored compliance with youth tobacco laws since January 1, 2002, by using youth monitors, who misreport their ages to attempt to purchase tobacco products.

A study testing compliance with youth bans for tanning beds in Melbourne, Australia, found that when young compliance monitors told attendants their true age, only 1 in 30 attendants allowed them access; when the monitors concealed their age or claimed to be 18 years old, 80% of attendants allowed them access (6). This finding highlights that results from standard compliance monitoring that employs protocols in which young compliance monitors are required to give their true age if asked do not accurately reflect levels of compliance under real-world conditions. Accordingly, it is important to employ protocols that more closely model the access attempts of youth outside of studies.

### Protect young people and other high-risk groups from tanning bed exposure

Given the increased risk of melanoma among those who first use a tanning bed before the age of 35 (3), restricting the age of first tanning bed use should reduce melanoma risk later in life. In addition to this restriction, legislation should restrict unsupervised operations where exposure is not supervised and should mandate comprehensive compliance monitoring.

Although regulations to restrict the sale of tobacco to minors are well established in most Western countries, effective regulations to control tanning bed use are rare, despite recommendations of the International Commission on Non-Ionizing Radiation Protection (ICNIRP) that people under 18 should not use tanning beds (13). Several jurisdictions now prohibit minors from using tanning beds (Box), but compliance with laws has been poor (14) or is in most cases either not tracked or not publically reported. In many countries, no laws regulate use of tanning beds.

Although tanning bed use is a health risk for all, it is a particular risk for people with fair skin, who are at high risk of negative health effects from tanning beds (15). However, only a few jurisdictions have implemented legislative controls to protect this high-risk group from tanning bed exposure. One exception is Brazil, where tanning beds are banned, an accomplishment unmatched with tobacco anywhere in the world. A sign that more outright bans of tanning beds may become likely is the recent commitment by several states of Australia (New South Wales, South Australia, and Victoria) to ban all tanning bed operations by the end of 2014.

### Offer alternatives to enable tanning bed users to quit

Although acceptance of a natural skin tone is the best public health outcome, social norms and predominant media imagery that suggest that a suntan is fashionable are difficult to fight (16,17). Alternative measures are available, such as spray-on tans and lotions that produce a tanned skin tone without the health risks associated with tanning bed use or sun tanning. Small studies of interventions that have promoted the use of sunless tanning products as an alternative to sunbathing or tanning bed use have shown positive results in reducing harmful tanning behaviors (18).

### Warn about the dangers of tanning bed use

Antitobacco public health campaigners have had tremendous success in leveraging research outcomes and public opinion data to help create newsworthy stories and influence opinions and policy. Paid mass media campaigns have played a vital role in informing the public about the dangers of tobacco use, changing social norms, and building support for policy reform around tobacco use (19). Experience in tobacco control efforts has shown that media information about the harmful effects of tobacco use is critical. For example, a study in New Zealand showed that tobacco sales declined with each increase in the number of articles on tobacco issues in daily newspapers (20). Effective dissemination of the evidence for the health risks of exposure to secondhand smoke, in particular, influences the number of smoke-free bylaws adopted by local governments (21).

We have also seen examples of the effect of increased awareness of the risks associated with tanning bed use on reducing demand for tanning beds. In 2007, a young Australian woman, Clare Oliver, died as a result of her very public battle with melanoma, which she attributed to her prior tanning bed exposure (22). The broad awareness of Oliver’s plight among the Australian population and the media commentary that followed increased perceptions of the risk associated with tanning bed use among the general population and triggered the introduction of legislation in several states. Following this combination of media coverage and legislation enactment, the percentage of tanning bed operators in some Australian cities decreased by as much as 51% (22). Similarly, in Denmark, tanning bed use has decreased concurrently with intensive media campaigns to raise awareness of the associated dangers, particularly among young people, and support for restricting minors’ admission to tanning beds has increased (23). Clarifying the societal costs of tanning bed use by such methods as analyzing the economic costs to health systems (5) could facilitate greater momentum in efforts to control the industry.

### Implement and enforce bans on advertising, promotion, and sponsorship

Regulations to restrict the promotion and advertising of tobacco now exist in most Western countries; however, the implementation of effective regulations to control tanning bed advertising and promotion is still in its infancy. Existing legislation on trade practices could compel the tanning bed industry to withdraw advertising that emphasizes health advantages and downplays the risks of skin damage and skin cancer.

In the United States, trade practice legislation through the Federal Trade Commission (FTC) has been used successfully in prosecuting both the tobacco and tanning bed industries (24,25). For example, in 1996, the FTC found that California Sun Care Inc had published false and misleading health claims in advertisements and promotional materials and required the company to pay for $1.5 million in corrective advertising (24). In 2010, the FTC issued a consent order that prohibited the Indoor Tanning Association (ITA) from making false health and safety claims about indoor tanning (25).

Also successful was a public interest lawsuit in which the Australian Competition and Consumer Commission alleged that the Australian Tanning Association Inc and 2 tanning bed franchise owners had engaged in false, misleading, and deceptive conduct related to the promotion of tanning bed use (26). The lawsuit further alleged that “solarium [tanning bed] distributors and operators who fail to warn customers of the health risks are in danger of breaching the Trade Practices Act.” This legal action resulted in injunctions against the 3 entities sued, restraining them from engaging in similar offending conduct, that is, from engaging in false, misleading, and deceptive conduct related to the promotion of tanning bed use (26).

### Raise taxes on tanning bed services

Increasing the price of the product through taxation is one of the most powerful ways to reduce tobacco consumption. For every 10% increase in cigarette prices, cigarette consumption can be expected to fall by approximately 4% (27).The United States introduced a tax on tanning bed services, a 10% excise tax, effective July 1, 2010. The effect, if any, of this tax on reducing demand is uncertain, although the percentage of young people in grades 9 through 12 who reported tanning bed use decreased slightly from 2009 through 2011; this decrease was not significant (28). Anecdotal evidence suggests that many tanning bed operators in the United States absorbed this 10% tax without increasing their prices to the consumer. More monitoring of the effect of increased prices on tanning bed use is required to determine whether taxes have a deterrent effect similar to what has occurred with tobacco.

## Looking Ahead

Lessons from the tobacco experience could influence the role of government in reducing demand for tanning beds. Restricting the use of tanning beds by those under the age of 18 and those with fair skin, increasing the price of services through taxation, licensing operators, and banning unsupervised operations are a few of the tools available to government that could reduce the harm associated with tanning bed use. However, regulations alone are unlikely to be sufficient unless jurisdictions monitor compliance, enforce legislation, and raise awareness of the dangers associated with tanning bed use. Also, legislation without education could have the opposite effect and increase demand if tanning bed users wrongly think that licensed tanning bed operations are safe because they are regulated by government.

The most important message from the antismoking experience is that tanning bed policy reform requires a sustained, well-resourced, multidimensional effort based on sound research. The experience of tobacco control advocacy suggests that public health advocates working to reduce the harm associated with tanning bed use must set realistic goals, derive satisfaction from partial victories, and commit for the long term. If we have learned anything from the fight against the tobacco industry, it is that victories in public health are rarely won easily or quickly.

**Box. Jurisdictions With Legislation Prohibiting People Under Age 18 From Using Tanning Beds**

**Table:** 

Jurisdiction	Year Effective
**Australia**	
Australian Capital Territory	2010
New South Wales^a^	2009
Northern Territory	2010
Queensland	2009
South Australia^a^	2008
Tasmania	2009
Victoria^a^	2009
Western Australia	2008
**Europe**	
Austria	2010
Belgium	2007
Finland	2012
France	1997
Germany	2009
Ireland	Pending (expected 2013)
Italy	2011
Norway	2012
Portugal	2005
Spain	2002
Sweden	Pending
**United Kingdom**	
England	2011
Northern Ireland	2012
Scotland	2009
Wales	2011
**Canada**	
British Columbia	2012
Newfoundland and Labrador (same province)	2012
Nova Scotia	2010
Ontario	Pending
Quebec	2012
**United States**	
California	2012
New York^b^	2012
Vermont	2012
**Other countries**	
Brazil^c^	2009
Iceland	2011

a Further legislation announced in 2012 that will ban all tanning beds for commercial use from December 31, 2014.

b Bans all persons under age 17 from accessing tanning beds.

c Bans all tanning beds for commercial use.
